# Segmental uncoverage ratio analysis of Crowe type-IV developmental dysplasia of the hip via 3-dimensional implantation simulation

**DOI:** 10.1186/s42836-020-00032-w

**Published:** 2020-05-19

**Authors:** Yiming Dou, Jianlin Xiao, Xinggui Wen, Jianpeng Gao, Hao Tian, Jianlin Zuo

**Affiliations:** Department of Orthopaedics, China-Japan Union Hospital of Jilin University, No. 126, Xiantai Street, Changchun, 130033 Jilin Province China

**Keywords:** Developmental dysplasia of hip, Total hip arthroplasty, Segmental uncoverage ratio, 3-D implantation simulation

## Abstract

**Background:**

To study the segmental uncoverage ratio (UCR) of a 44-mm cup model placed in a true acetabulum of Crowe type-IV developmental dysplasia of the hip via 3-Dimensional (3D) implantation simulation.

**Methods:**

Qualified CT imaging data of 26 patients (involving 30 hips) with Crowe type-IV DDH were imported into Mimics software for reconstruction. Then a 44-mm eggshell cup model was placed in a true acetabulum. First, total uncoverage ratio (TUCR) was measured. Then the virtual cup was divided into 4 segments according to the quadrant setting of the true acetabulum, i.e., anterior-superior (A-S) segment, anterior-inferior (A-I) segment, posterior-superior (P-S) segment and posterior-inferior (P-I) segment. The UCRs of the aforementioned segments were measured, i.e., anterior-superior uncoverage ratio (A-SUCR), anterior-inferior uncoverage ratio (A-IUCR), posterior-superior uncoverage ratio (P-SUCR) and posterior-inferior uncoverage ratio (P-IUCR). The acetabular height and anterior-posterior diameter on the 3-D model were also calculated. Statistic analyses were performed by using SPSS software package.

**Results:**

TUCR was 0.2958 ± 0.1003 (95% [CI], 0.1020 to 0.5400) in this cohort of Crowe Type-IV hips. P-SUCR had the greatest value among all the segmental UCRs (0.1012 ± 0.0435, 95% confidence interval [CI],0.0152 to 0.1914) and the most significant positive correlation with TUCR (Pearson correlation = 0.889, *p* < 0.01. Linear regression R^2^ = 0.791). Similarly, P-IUCR and A-SUCR showed a significant positive correlation with TUCR. However, A-IUCR exhibited no correlation with either total or other segmental UCRs. P-SUCR was found to bear significant positive correlation with P-IUCR (pearson correlation = 0.644, *p* < 0.01. Linear regression R^2^ = 0.415). Acetabular height and A-P diameter were not correlated with TUCR.

**Conclusion:**

Implantation of a 44-mm cup into Crowe type IV acetabulum is feasible and could achieve acceptable host bone coverage in most of the cases. P-SUCR contributed most to TUCR. TUCR had no linear relationship with the size of the host acetabulum, suggesting that the pre-operative plan should be individualized.

## Introduction

Developmental dysplasia of the hip (DDH) represents the most common cause leading to secondary osteoarthritis of hip. As the disease progresses, patients may develop such symptoms as hip pain, limitation of movement, which can seriously affect the quality of life of the patients [1–3]. For severe DDH, total hip arthroplasty (THA) is an effective treatment which can relieve pain and help the join regain function. DDH is morphologically classified in different ways, including the Crowe classification and the Hartofilakidis method [4, 5]. Due to the complexity of acetabular anatomy, the treatment of the acetabulum varies widely. Different operative principles apply with different classifications, and even within one classification, surgery strategies vary. Among those classifications, Crowe type-IV acetabula are often small and shallow, irregular in shape, and may be associated with bone defects. Mulroy and Harris *et al* mentioned that, in order to prevent mechanical graft failure, a 70% coverage of the cup by host bone is recommended [6]. With Crowe type-IV DDH, it is an feasible choice to use a 44-mm cup to achieve a balance between small bone volume in a true acetabulum and adequate cup size for hip stability. So far, few three-dimensional studies focused on the uncoverage ratio of this type of DDH. Our research questions are as follows: What are the segmental uncoverage ratios of a 44-mm cup placed in a true acetabulum in Crowe type-IV DDH and what are their influencing factors? We aimed to address these questions via three-dimensional implantation simulation.

## Materials and methods

### Study subjects

We retrospectively reviewed the preoperative imaging data of 115 patients with DDH who were admitted to our institution from January 2010 to August 2018. The patients were categorized according to the Crowe classification. Of them, 30 subjects (35 hips) were classified as Crowe type-IV. In 2 of the 30 patients, the imaging results were not satisfactory, and 2 had abnormal pelvic morphology. These 4 patients (5 hips) were excluded. Eventually, 26 Crowe type-IV patients (30 hips) were included in our study. Demographic data of the subjects are shown in Table 1.

**Table 1:** Demographic data

	Hip	Male/Female	Age^a ^(yr)	Height^a ^(cm)	Weight^a ^(kg)	BMI^a ^(kg/m^2^)
Crowe IV DDH	30	2:24	41.85 ± 13.14 (22 to 70)	156.75 ± 11.03 (141 to 175)	57.38 ± 13.08 (33 to 84)	23.42 ± 5.63 (12.12 to 27.43)

^a^The values are expressed as the mean and the standard deviation, with the 95% confidence interval in parentheses. *BMI* Body mass index

### CT scan and 3-D reconstruction

**Fig. 1:**
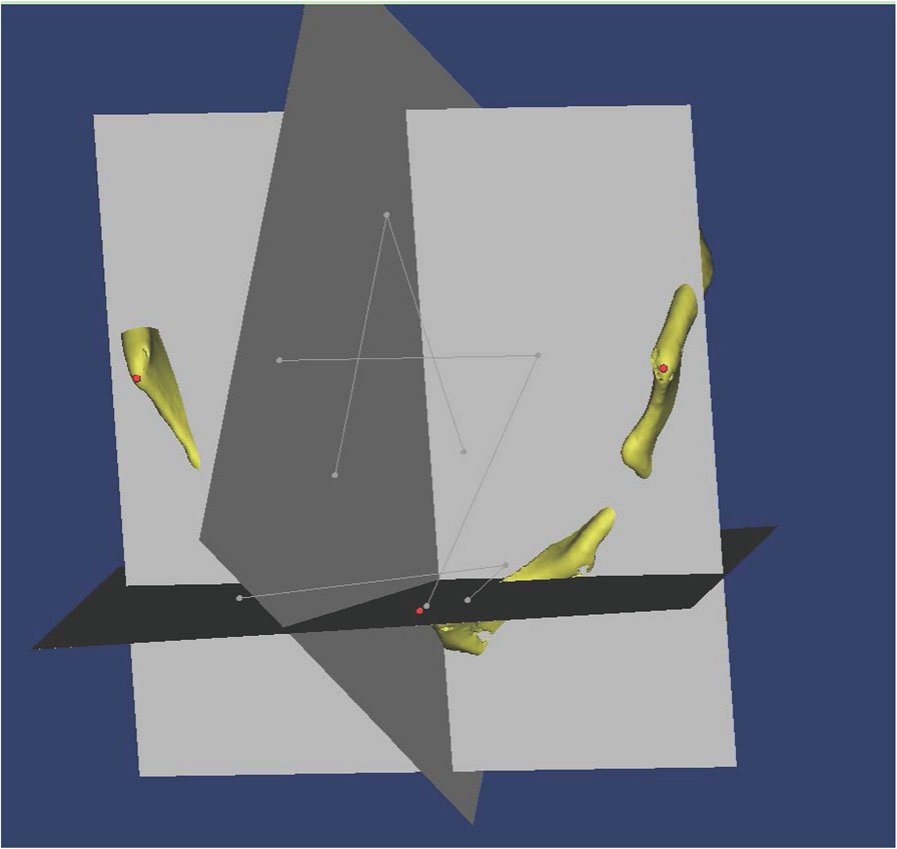
The coronal plane, sagittal plane and horizontal plane of the pelvis

Pelvic CT was performed with a Toshiba Aquilion CT scanner (120 kV, 320 mA, 512 × 512 matrix, and 0.5-mm slice thickness). The patients were placed in a supine position with the patellae facing the ceiling. Scan was performed from the iliac crest to the distal one-third of the femur. All CT slices were saved in Digital Imaging and Communications in Medicine (DICOM) format and imported into Mimics software (Version 19.0, Materialise) for 3-D reconstruction. Before measurement, we used the same method to set our standard planes as previously reported [7]. Bilateral anterior-superior iliac spines (ASIS) and pubic tubercles were identified and the coronal plane was established. The sagittal plane was perpendicular to the coronal plane while passing through the mid-point of pubic tubercles, and the horizontal plane was perpendicular to the coronal plane and the sagittal plane. Thus the standard planes were set for the following measurements. Those standard planes are showed in Fig. 1.

### 3-D implantation simulation of cup model

We created 2 hemispherical virtual acetabular cup models by means of a 3-matic software package (Version 9.0, Materialise). One was a virtual cup model with an outer diameter of 44-mm and a shell thickness of 4 mm. Then we performed cup implantation by using the virtual cup model. The other was also a virtual cup component with the same outer diameter but its shell thickness was only 0.01 mm. The model served as an eggshell cup for measuring the uncoverage area. These 3-D models were imported into the Mimics software (Version 19.0, Materialise) in STL (stereolithography) format. In THA patients without hip dysplasia, the cup was selected according to the actual size of the acetabulum. While in THA patients with Crowe type IV hip dysplasia, the bone volume in a true acetabulum was too limited to accommodate a larger cup. In most inventories of the hip prostheses, a 44-mm diameter cup is the smallest one to accommodate a 28-mm head to ensure better range of motion and to lower risk of impingement and dislocation. The diameter of most of the true acetabulum in this type of hip is less than 40 mm. Thus, it is an feasible choice to use a 44-mm cup to accomplish a balance between a small bone volume in true acetabulum and adequate cup size for hip stability.

The following were the rules of our virtual cup replacement. All cup models were implanted into a true acetabulum at an abduction angle of 45° ± 1° and an anteversion angle of 20° ± 1°. We medialized the cup to make it abut the medial wall to maximize the coverage by host bone. The front edge of cup was tangent to the anterior wall of the true acetabulum, and the lower edge of the cup was tangent to the transverse ligament. Due to the bigger size of the cup, as compared to the size of the true acetabulum, the rotational center would slightly migrate posterior-superiorly. All 44-mm standard cups could be successfully implanted into the true acetabulum with this method. Then we replaced the standard cup model with the eggshell cup model and the uncoverage area was calculated according to the uncovered area of eggshell cup.

### 3-D acetabular quadrants set and measurements of segmental UCRs

**Fig. 2:**
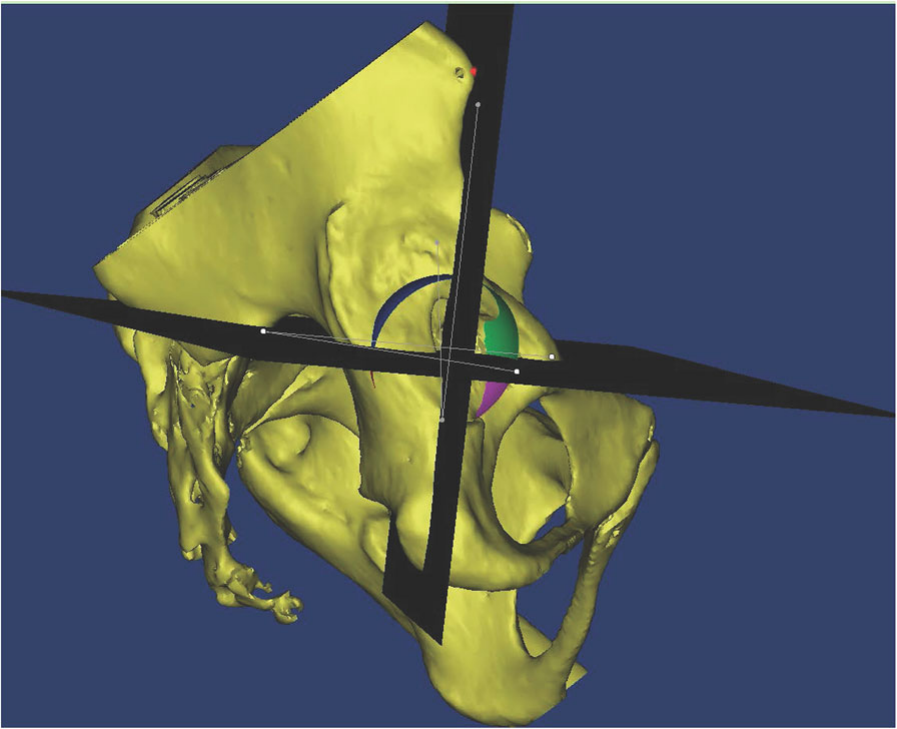
The acetabular quadrants divided the cup into 4 segments: anterior-superior (AS), anterior-inferior (AI), posterior-superior (PS), posterior-inferior (PI)

**Fig. 3:**
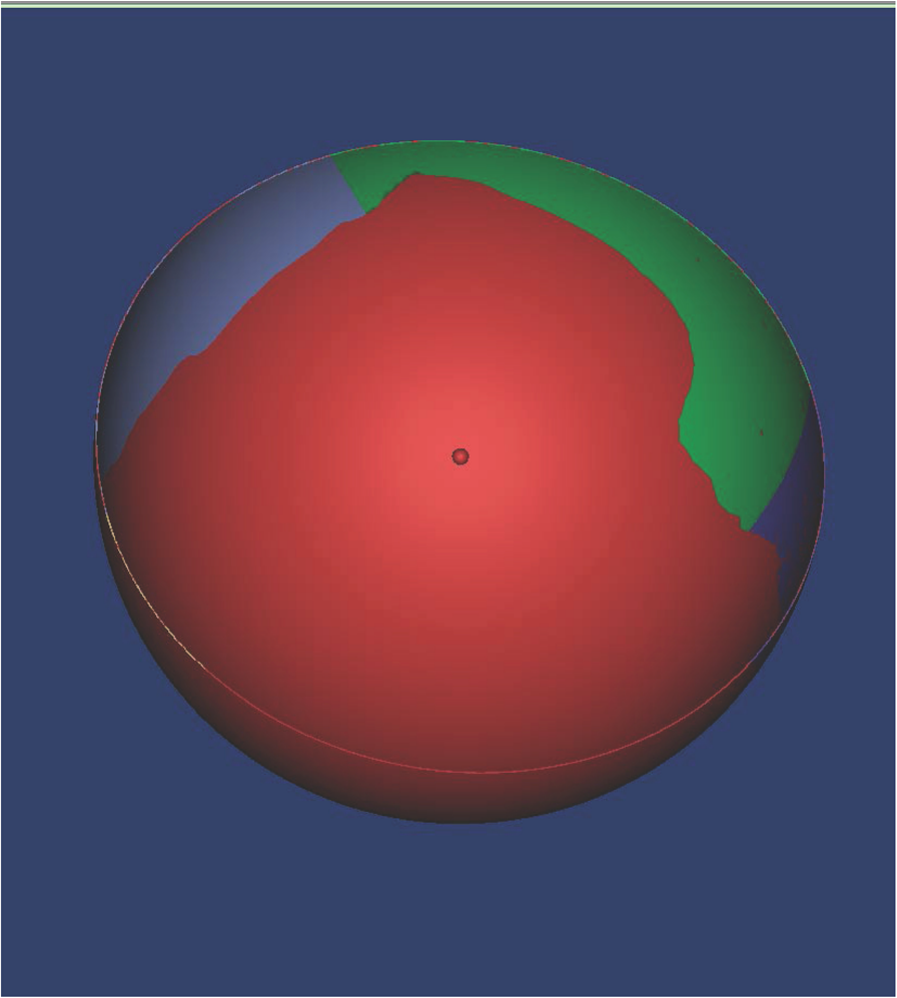
The uncoverage area of each segments, generated by Mimics software

Wasielewski RC *et al* divided acetabulum into 4 quadrants in order to find the safe zones for screw fixation [8]. In 2-dimensional image, we drew a line passing through the ipsilateral ASIS and the center of cup as y-axis, and the line passing the center of cup while perpendicular to y-axis served as x-axis. In our study, we designated the plane passing through bilateral ASIS and the center of the implanted virtual cup the acetabular quadrantal sagittal plane, and the plane perpendicular to acetabular quadrantal sagittal plane, going through the cup center, was designated acetabular quadrantal horizontal plane. These 2 planes divided the cup into 4 segments: anterior-superior (A-S), anterior-inferior (A-I), posterior-superior (P-S), posterior-inferior (P-I) segments (Fig. 2). We calculated the uncoverage area of each segment by using the Mimics software (version 19.0, Materialise), and the uncoverage ratio equaled uncoverage area divided by total surface area. (Fig. 3).

### Measurements of acetabular height and anterior-posterior diameter

We used Mimics software (Version 19.0, Materialise) to get the coordinates of the most superior point of the true acetabulum and establish the horizontal plane at the level of acetabular lower edge. We applied Matlab software (Version 2016, Mathworks) to calculate the perpendicular distance from the most superior point to the horizontal plane with the following formula: D = abs(a*p [1]+b*p [2]+c*p [3]+d)/sqrt(a^2 + b^2 + c^2). Then we placed horizontal plane at the level of 1/2 acetabular height, and the distance from the anterior wall to the posterior wall was A-P diameter of the true acetabulum. In this cohort, the true acetabula was of conic shape, and the standard volumetric formula was V = 1/3*π*r^2^*H. We assumed that there might be a proportional relation between r and A-P diameter, so we calculated the volume of the true acetabulum with the following formula: V=H*R*R (where H is acetabular height; R is A-P diameter).

### Statistical analysis

All statistical analyses were performed by the same author using a statistical software package (SPSS Version 20, SPSS Inc., Chicago, IL, USA). The Kolmogorov-Smirnov test was conducted to find out the distribution pattern of all data. The Pearson test and linear regression analysis were performed to estimate the correlations among UCRs; height, A-P diameter and volume of the acetabulum. The scatter diagrams were plotted to illustrate the correlation pattern of the former data. All data were expressed as mean and standard deviation (SD). All reported *P*-values were 2-tailed and the significance level was set at 0.05.

## Results

The results of UCRs were as follows: 0.0832 ± 0.0427 (95% [CI], 0.0303 to 0.1667) for A-SUCR, 0.0521 ± 0.0246 (95%[CI], 0.0013 to 0.1255) for A-IUCR, 0.1012 ± 0.0435 (95% [CI], 0.0152 to 0.1914) for P-SUCR, 0.0592 ± 0.0478 (95% [CI], 0 to 0.1568) for P-IUCR, and 0.2958 ± 0.1003 (95% [CI], 0.1020 to 0.5400) for TUCR. The mean acetabular height was 31.99 ± 4.09 mm (95%[CI], 24.44 to 40.55 mm). The mean A-P diameter was 25.23 ± 4.82 mm (95%[CI], 14.65 to 40.00 mm). The mean volume was 21,257.59 ± 9165.69 (95%[CI], 5785.17 to 54,070.88 mm^3^). Data are detailed in Table 2.

**TABLE 2:** Statistical results

	Measurement	Pearson correlation & Linear regression results							
		ASUCR	AIUCR	PSUCR	PIUCR	TUCR	H	R	V
ASUCR*	0.0832 ± 0.0427 (0.0303 to 0.1667)	–	–	–	–	0.461^a^ R^2^ = 0.213	–	–	–
AIUCR*	0.0521 ± 0.0246 (0.0013 to 0.1255)	–	–	–	–	–	–	–	–
PSUCR*	0.1012 ± 0.0435 (0.0152 to 0.1914)	–	–	–	0.644^b^ R^2^ = 0.415	0.889^b^ R^2^ = 0.791	–	–	–
PIUCR*	0.0592 ± 0.0478 (0 to 0.1568)	–	–	–	–	0.768^b^ R^2^ = 0.590	–	–	–
TUCR*	0.2958 ± 0.1003 (0.1020 to 0.5400)	–	–	–	–	–	–	–	–
H*	31.99 ± 4.09 (24.44 to 40.55)	0.395^a^ R^2^ = 0.156	–	–	− 0.585^b^ R^2^ = 0.342	–	–	–	–
R*	25.23 ± 4.82 (14.65 to 40.00)	–	–	–	–	–	–	–	–
V*	21,257.59 ± 9165.69 (5785.17 to 54,070.88)	–	–	–	−0.473^b^ R^2^ = 0.224	–	–	–	–

*AS* Anterior-superior, *AI* Anterior-inferior, *PS* Posterior-superior, *PI* Posterior-inferior, *UCR* Uncoverage ratio, *H* Acetabular height, *R* Anterior-posterior diameter, *V* V=H*R*R, V as acetabular volume

^a^*p*<0.05. ^b^*p*<0.01

### The relationship between acetabular size and UCRs

Pearson correlation and linear regression analysis showed that the acetabular height bore a significant negative correlation with P-IUCR (Pearson correlation = − 0.585, *p* < 0.01. Linear regression R^2^ = 0.342) and a significant positive correlation with A-SUCR (Pearson correlation coefficient = 0.395, *p* < 0.05. Linear regression R^2^ = 0.156). The acetabular height exhibited no correlation with other UCRs. The A-P diameter displayed no correlation with all UCRs. The acetabular volume had a significant negative correction with P-IUCR (Pearson correlation coefficient = − 0.473, *p* < 0.01. Linear regression R^2^ = 0.224), but was not correlated with other UCRs. The mean TUCR was 0.2958 ± 0.1003 (95%[CI], 0.1020 to 0.5400). Twenty TUCRs≤0.3 (mean acetabular height: 32.86 ± 4.18 mm, from 26.38 to 40.55 mm. mean A-P diameter: 25.00 ± 4.13 mm, from 14.65 to 31.55 mm), while the other 10 TUCRs> 0.3 (mean acetabular height: 30.67 ± 3.75 mm, from 24.44 to 35.97 mm. mean A-P diameter: 25.58 ± 5.89 mm, from 17.53 to 40.00), of which 4 TUCRs> 0.4. If we designated those TUCRs> 0.3 group A, and TUCRs≤0.3 group B, there were no significant differences in height and A-P diameter of the true acetabulum between the two groups.

### The relationship between each segmental UCR and TUCR

P-SUCR showed a significant positive correlation with P-IUCR (Pearson correlation coefficient = 0.644, *p* < 0.01. Linear regression R^2^ = 0.415). P-SUCR exhibited a significant positive correlation with TUCR (Pearson correlation coefficient = 0.889, *p* < 0.01. Linear regression R^2^ = 0.791), so did P-IUCR and A-SUCR (Pearson correlation coefficient = 0.768, *p* < 0.01. Linear regression R^2^ = 0.590 for P-IUCR and Pearson correlation coefficient = 0.461, *p* < 0.01. Linear regression R^2^ = 0.213 for A-SUCR). A-IUCR showed no correlation with TUCR.

## Discussion

This study showed that the mean TUCR was 0.2958 ± 0.1003 (95%[CI], 0.1020 to 0.5400), which means implanting a 44-mm cup into a true acetabulum is workable in most Crowe type-IV DDH hips. Nonetheless, with some hips, TUCRs are more than 0.3 and some surgical techniques should be used with these hips to secure adequate coverage ratio, such as structural bone grafts, high placement of cup, extra-small cup, posterior-superior placement of cup, medial protrusio technique, among others [9–11] These techniques, however, may negatively impact the long-term survival of the implants. Previous studies revealed that the long-term failure rate of structural bone grafts was high (39–75%) [6, 11, 12], and using extra-small cup would decrease femoral head-neck radio and thereby increase shear stress. High placement of cup may compromise the biomechanical property and then shorten the long-term survival of the hip [13]. This study demonstrated that no significant difference was found in the height, A-P diameter and volume of the true acetabulum between the TUCR 0.3 minus group and the TUCR 0.3 plus group. This finding has two implications. First, surgeons must make individualized plans for each single DDH patient, because larger true acetabulum does not mean better coverage. Second, host bone stock in the true acetabulum is not quantitatively related to the size of the true acetabulum. Hence, a 44-mm cup is not contraindicated for a small true acetabulum.

As for the placement location of cup, Hartofilakidis *et al* proposed that surgeons should ream towards posterior-superior direction, because bone stock in that direction is usually adequate [14]. Sen C *et al* and Greber EM *et al* indicated that the bone stock of the posterior column is sufficient while the bone stock of the anterior-superior wall is insufficient [15, 16]. Dorr *et al* proposed a medial protrusio technique which could maximize host bone coverage to the cup [17]. Dunn *et al* mentioned a medial wall breakthrough technique which could further maximize host bone coverage to the cup [9]. In our study we found that 10 TUCRs> 0.3, of which 4 TUCRs> 0.4. Thus it is reasonable to use one of the previous techniques to enhance coverage of host bone to the cup when the uncoverage ratio is unacceptable.

Few studies so far analyzed the segmental uncoverage ratio of the cup of Crowe type IV DDH. We believe that each segment contributes, to various degrees, to the total coverage. Out study showed that the mean P-SUCR was 0.1012 ± 0.0435 and bore the most significant positive correlation with TUCR, suggesting that P-SUCR affects TUCR most. Given that P-SUCR is very important during cup insertion, when P-SUCR is seriously inadequate we should consider structural bone grafting or other coverage-enhancing techniques.

This study had some limitations. First, there were too few male subjects (2 males, while 24 females). As a result, conclusion should be applied to male Crowe type-IV patients with discretion. Second, the number of subjects in this cohort was relatively small, this might affect the significance of differences in some parameters, such as the uncoverage ratio and true acetabulum size. Third, we set the anterior pelvic plane as the standard plane without involving the pelvic tilt angle and this omission might influence the functional acetabular anteversion during operation. Fourth, the 44-mm cup was chosen for all patients in this study. Some Crowe type IV hips may accommodate 44-mm+ cups, although the diameter of most of the true acetabula in this type of hip is less than 40 mm.

## Conclusion

Our study showed that implantation of a 44-mm cup in Crowe type IV acetabulum was feasible and could achieve acceptable host bone coverage in the majority of cases. For those patients with an unacceptable uncoverage ratio, it is reasonable to perform structural bone grafting or other coverage-enhancing techniques. P-SUCR contributed most to TUCR. TUCR bore no linear relationship with the size of the host acetabulum, suggesting that the preoperative plan should be individualized.

## Data Availability

The datasets used and/or analysed during the current study are available from the corresponding author on reasonable request.

## Abbreviations

DDH: Developmental dysplasia of the hip
UCR: Uncoverage ratio
TUCR: Total uncoverage ratio
A-SUCR: Anterior-superior uncoverage ratio
A-IUCR: Anterior-inferior uncoverage ratio
P-SUCR: Posterior-superior uncoverage ratio
P-IUCR: Posterior-inferior uncoverage ratio
STL: Stereolithography
